# Primary healthcare providers’ strategies of handling patients presenting with potential cancer symptoms following the introduction of standardized cancer patient pathways in Sweden

**DOI:** 10.1186/s12875-026-03600-2

**Published:** 2026-10-10

**Authors:** Petter Fjällström, Anna-Britt Coe, Mikael Lilja, Senada Hajdarevic

**Affiliations:** 1 Department of Nursing, Umeå University, Umeå, SE-901 87 Sweden; 2 Department of Social and Psychological Studies, Karlstad University, Karlstad, SE-651 88 Sweden; 3 Department of Public Health and Clinical Medicine, Unit of Research, Education, and Development, Östersund Hospital, Umeå University, Umeå, Umeå, SE-901 87 Sweden; 4 Department of Public Health and Clinical Medicine, Family Medicine, Umeå University, Umeå, SE-901 87 Sweden

**Keywords:** Cancer patient pathways, Cancer symptoms, Primary healthcare, Professional competencies, Standardization, Timely diagnosis

## Abstract

**Background:**

Early cancer diagnosis is a healthcare priority, but identifying patients who may have cancer remains challenging because alarm symptoms are often non-specific and may also indicate other conditions. To facilitate timely cancer diagnosis, standardized cancer patient pathways (CPP) have been introduced in several health systems, including Sweden. Since most patients diagnosed with cancer begin their pathway in primary healthcare (PHC), nurses and physicians play a central role in assessing symptoms and determining whether CPP criteria for suspected cancer are fulfilled. Although research concerning the introduction of CPPs is expanding, knowledge is lacking on how CPPs influence Swedish PHC nurses’ and physicians’ ways of handling patients with potential cancer symptoms. Therefore, this study aimed to explore PHC providers’ strategies of using CPPs when handling patients presenting symptoms that may indicate cancer.

**Method:**

A constructivist Grounded Theory Method was utilized to collect and analyze qualitative data through group interviews in PHC units in northern Sweden. Six units were included, and nine group interviews were conducted from March to November 2019, involving 21 nurses and 20 physicians. Interviews were audio recorded, transcribed verbatim, and analyzed.

**Results:**

The results showed a process of handling challenges and opportunities when suspecting cancer in PHC and using CPPs through three categories focusing on strategies. First, physicians and nurses prioritized patients’ needs of urgent care through conviction by using their professional competence to assess symptoms, rather than strictly relying on the symptom descriptions outlined in CPPs, with nurses primarily identifying these symptoms. Second, while physicians used creative strategies to make CPP referrals work for patients whose symptoms did not align with the templates, nurses similarly used creative strategies to raise suspicions and facilitate further assessment despite the absence of clear alarm symptoms. Third, physicians and nurses sensitively informed patients about CPPs, balancing between explaining the next investigation steps and safeguarding potential illness-related anxiety by avoiding the discussion as much as possible.

**Conclusion:**

Findings highlight the important role of PHC providers in using CPPs for patients with potential cancer by identifying symptoms, investigating suspicions, and sensitively informing patients. CPPs were perceived as facilitating faster cancer diagnostic pathways when they aligned with PHC providers’ clinical assessments, but challenges arose when clinical assessments misalign with predetermined standardized packages. These findings indicate the importance of involving PHC providers in refining CPP usage. Furthermore, communicating clearly about potential cancer diagnoses is necessary, but not as simple as it may seem.

**Supplementary Information:**

The online version contains supplementary material available at https://doi.org/10.1186/s12875-026-03600-2.

## Background

Early cancer diagnosis is associated with improved survival, better clinical outcomes, and increased opportunities for treatment [1, 2]. Timely cancer diagnosis has become a priority in health systems, prompting efforts to optimize diagnostic pathways and reduce delays in the diagnostic process [3, 4]. However, early cancer diagnosis is difficult because most of these alarm symptoms are frequently perceived even in other conditions, and cancer may not necessarily present with such symptoms [5, 6]. Consequently, it is necessary to act effectively and without delay to identify, among all patients seeking care, precisely those who might have cancer.

Therefore, health systems increasingly standardize healthcare provision by adopting uniform practices across time and space through agreed rules [7] as a way to promote efficiency, support evidence-based practice, and reduce variation in healthcare delivery [8, 9]. Cancer patient pathways (CPP) in Denmark [10] and the Two-Week Wait pathway in the UK [11] are examples of such standardization and have been introduced in health systems to facilitate timely cancer diagnosis. CPPs comprise a standardized set of predetermined time frames, examinations, and procedures focused on guiding healthcare providers to treat patients with symptoms that can indicate cancer to improve patient prognosis and the quality of care [2, 10]. Earlier experiences from Denmark and the UK suggest that CPPs have contributed to shorter diagnostic intervals and may be associated with improved outcomes [11, 12]. During the last decade, Nordic countries including Sweden have adopted CPPs to reduce unacceptable waiting times for cancer diagnosis and treatment [13]. In northern Sweden, long geographical distances and sparsely populated areas may pose additional challenges to timely cancer diagnosis and referral, making it a particularly relevant setting for exploring the use of CPPs.

In Sweden, CPPs start with the presence of a well-founded suspicion of cancer based on alarm symptoms, tests, or indications that suggest a significant risk of cancer, and end either upon the start of treatment or the rejection of the cancer suspicion [14]. Over 70% of patients newly diagnosed with cancer start their cancer pathway in primary healthcare (PHC) units [15]. PHC providers interpret symptoms and test results and determine whether they are alarming and the criteria for cancer suspicion are met, thereby guiding referral to cancer care services [16, 17]. Thus, they often act as gatekeepers before patients are referred for further investigation [16, 18]. Research on early cancer diagnosis within the PHC setting has predominantly focused on PHC physicians’ perceptions concerning timely cancer diagnosis [19, 20]. Meanwhile in Swedish PHC, nurses together with physicians are the first healthcare professionals to encounter patients seeking care [21] and therefore play an important role in the pathways to cancer diagnosis.

Research concerning CPPs generally indicates a reduction in patient’s time to diagnosis [10, 11], although this effect is not observed for all patient groups [2, 22]. Despite growing research on healthcare providers’ experiences with standardization in their daily work, there remains a lack of research addressing the influence of CPPs in PHC settings. For example, Wells et al. [23] found that while CPPs help PHC physicians streamline their work, concerns have been raised that CPPs lack clarity on the specific investigations before initiating a CPP referral. Although research concerning the introduction of CPPs is expanding, knowledge is missing on how this may influence Swedish PHC nurses’ and physicians’ ways of handling patients with potential cancer symptoms.

## Aim

To explore primary healthcare providers’ strategies of using CPPs when handling patients presenting symptoms that may indicate cancer.

## Method

### Design

The study employed a constructivist Grounded Theory Method (GTM) design according to Charmaz [24] to gather and analyze qualitative data through group interviews. Constructivist GTM enabled us to explore the actions and meanings that healthcare providers assign to handling potential cancer symptoms after the introduction of CPPs in PHC units and to generate conceptual categories grounded in participants experiences. The study was guided by the Consolidated Criteria for Reporting Qualitative Research (COREQ) 32-item checklist [25].

### Setting, recruitment, and participants

This study was carried out among PHC units within the health system in northern Sweden, where PHC often serves as the initial point of contact for patients presenting with symptoms that may indicate cancer. We began by purposively sampling PHC units from urban and sparsely populated areas, aiming to achieve variation in unit size and geographical location. Six PHC units (two in urban and four in sparsely populated areas) that were approached agreed to participate and were recruited into the study. Next, in each PHC unit, we purposively sampled physicians and nurses, as these professions are typically the first point of contact for patients needing cancer-related healthcare in Sweden. The inclusion criteria were being a physician or nurse currently working in PHC and having at least six months of PHC experience. Recruitment aimed to include participants with varying age, gender, professional experience, and experiences of working within PHC settings. The study’s purpose and methods were presented during a meeting at the PHC units. Subsequently, letters were sent to the managers of the respective PHC units to arrange appointments with the participating healthcare providers for interviews. These letters included detailed information regarding voluntary participation terms and informed consent. Nurses and physicians were then recruited from each participating PHC unit.

### Data collection

Data were collected in group interviews by three interviewers (SH, ABC, and a researcher previously working on this project) to explore shared and contrasting viewpoints [26], and each interview was facilitated by two of them; one acted as a moderator, and the other wrote field notes to stimulate participant interactions within each group [27]. The number of participants in each group ranged from two to eight (mean 5), depending on the practical considerations within each PHC unit during data collection. In three larger PHC units, more participants were available, allowing the separation of nurses and physicians into distinct interview groups for discussion (three groups each). However, in three smaller PHC units, interviews involved two separate groups including a mix of nurses and physicians, and one group exclusively encompassed nurses due to the shortage of physicians caused by other unplanned reasons of physicians on that day. The interviews were conducted between March and November 2019, encompassing nine groups including 21 nurses (all women) and 20 physicians (50% women), representing diverse work experience levels and urban and sparsely populated areas. An overview of participants’ work experiences (Table 1) and areas where data were collected (Table 2).

**Table 1:** Participants´ work experiences

Years of experience in primary healthcare settings	Nurses	Physicians
< 1 year	4	4
1–4 years	4	7
5–10 years	3	4
> 10 years	10	5

**Table 2:** Areas where data was collected

Group interviews (*n*)	Urban areas (> 10 000 listed patients)	Sparsely populated areas (≤ 10 000 listed patients)
Only nurses	2	2
Only physicians	2	1
Mixed including physicians and nurses		2

A semi-structured interview guide (Appendix 1) with open-ended questions was utilized during interviews with healthcare providers. The guide explored the healthcare providers’ approaches to patients presenting symptoms that may indicate cancer, their strategies for handling these symptoms, the influence of CPPs on their approach to addressing these symptoms, and how CPPs were perceived to influence their work. Throughout data collection, we added new questions regarding emerging categories. All interviews were conducted at the PHC units, during participants’ working hours, and audio recorded. During the interviews, moderators actively engaged all participants to express themselves on different issues. The interviews, ranging from 37 to 56 min (mean 46), were transcribed verbatim and then imported into MAXQDA 2018, a software program designed for data coding and analysis.

### Analysis

Throughout the data analysis process, we employed the coding techniques of the GTM, utilizing constant comparison between empirical data, codes, and memos [24]. We started with the initial coding phase of the group interviews, during which we generated memos to engage ourselves in the data and stimulate analytical ideas. We coded transcribed interviews line by line, interacted with each data fragment, and employed active codes to focus on actions and meanings. After the initial coding, we conducted the focused coding phase, organizing the initial codes into categories. We iteratively moved between these coding phases throughout the process, utilizing constant comparison to analyze and conceptualize the codes into categories and their interrelationships. Throughout the analysis, we also compared codes and categories across nurse-only, physician-only, and mixed professional groups to assess whether the emerging categories were presented across different group compositions. All three categories were identified across the different group types, although some aspects were expressed with varying emphasis related to the specific professional role. During the theoretical coding phase, we used the Theoretical Code Family of Strategies [28] to analyze participants experiences and construct strategies of using CPPs when healthcare providers handle suspicion of cancer in PHC settings. The analysis was performed by a multidisciplinary research team with backgrounds from nursing, medicine, and social sciences. Emerging codes, categories, and analytical memos were discussed regularly between authors while maintaining an open-minded approach firmly grounded in the empirical data. When analyzing the last 2 group interviews collected, no substantially new codes or category properties emerged, and no new interviews were conducted. Even if the first author (PF) conducted the coding, memo-writing, and category development, the research team continued together to analyze data using constant comparison between data, codes, categories, and memos, and found that these subsequent interviews primarily contributed to confirming the developing categories. The emerging codes, categories, were discussed within the research team throughout the whole analysis process to consider alternative interpretations and refine category development. When different interpretations arose, they were resolved through continued discussion and by returning to the original interview transcripts, codes, and memos to ensure that the analysis remained grounded in the data. All authors contributed to and agreed upon the interpretation of findings and the refinement of the final categories and their relationships.

## Results

Our results showed that in situations when suspecting cancer in PHC settings a process unfolded of handling a lot of challenges but also some possibilities for the providers to use CPPs to improve early cancer diagnosis. These challenges and possibilities comprise three categories focusing on three strategies of professional assessments and conviction to prioritize patients’ need of urgent investigation and care, creativity to make referrals work, and sensitivity to inform about starting CPPs when suspecting cancer (see Table 3).

First, when prioritizing patients’ need of urgent investigation and care among all patients contacting PHC units, CPPs were challenging nurses’ and physicians’ conviction and competence in determining the seriousness of symptoms. To decide the urgency of care, to some extent PHC physicians and nurses mainly relied on their professional competency while sometimes following the formal symptom descriptions in the CPP template. Second, in the next step, when clinically assessing the suspicion of serious diseases, such as cancer, the CPP templates challenged the physicians’ creativity to make referrals for investigations to work in line with CPP’s requirements. Even though the CPP template could facilitate PHC providers through their investigation process, this also required the creation of workarounds from the templates. Lastly, PHC providers’ sensitivity to inform about cancer suspicion was challenged since they sometimes choose to inform patients about undertaking CPPs and the next steps while occasionally they were avoiding it and thereby safeguarding patients from potential illness-related anxiety. The three categories are described next.

**Table 3:** A process of strategies to handle challenges and possibilities when using CPPs in PHC

Prioritizing the need of urgent care through conviction	Relying on professional competency
	Relying on formal symptom descriptions
Creatively making referrals work when suspecting cancer	Facilitating clinical investigations with the template
	Finding workarounds from the template
Sensitively informing about cancer suspicion	Telling patients what happens next with honesty
	Safeguarding patients by avoidance

### Prioritizing the need of urgent care through conviction

This category encompasses the PHC providers’, mainly nurses’, task of acting as the main entryway for patients contacting PHC units with symptoms indicating potential cancer. Challenges arose when patients presented such symptoms during their first point of contact, and PHC providers had to prioritize which patients needed urgent investigation and care the most, relying on their conviction—that is, their professional competencies, intuition, and clinical experience—rather than strictly following formal symptom descriptions.

PHC physicians and nurses often relied on their professional competencies to decide what further healthcare provision patients needed. Physicians were responsible for deciding whether to refer patients to cancer care through CPPs. However, it was primarily the nurses’ task to recognize alarm symptoms during the patient’s first contact, assess these symptoms seriousness, and prepare for further actions at the PHC unit. Physicians expressed a reliance on their own and nurses´ professional competence. They trusted in nurses’ competencies to prioritize patients presenting potential cancer symptoms, since nurses were most often the first point of contact in PHC units. For example, in a group interview, physicians emphasized this trust like this:

We trust the nurses’ professional knowledge and understanding of what could be cancer and needs to be scheduled quickly or what can wait, and they have to deal with this in a limited time… / Then, it is important that they (the nurses) have experience and can make their assessments.

(Group interview only physicians nr 2)

Meanwhile, nurses described the challenges of detecting potential cancer based on patient stories about their symptoms and concerns regarding potential illness. They emphasized how their daily work involved handling a wide range of symptoms, which helped them develop an alertness to signs of possible cancer and prioritizing this, as depicted in the next quote:

It’s important to be alert when you’re on the telephone counseling. Patients may call in with alarming symptoms that they don’t say clearly, but by asking a few more questions, they can tell you what kind of problem they have. There may be a patient who has never reached out to us before, but you get the sense that something makes them call today.

(Group interview only nurses nr 2)

The nurses were perceptive and asked the patients more in-depth, which was vital when they recognized that patients underestimated their symptoms’ seriousness, and using their intuition when a higher possibility of serious diseases such as cancer was perceived.

In addition to relying on professional competencies, PHC providers also relied on formal symptom descriptions outlined in the CPP templates. Nurses described the difficulty of assessing seriousness of symptoms in patients who frequently sought healthcare or exaggerating their symptoms. Despite these challenges, nurses prepared further actions using their professional competencies and played a central role in enabling patients to access continued healthcare. Physicians in turn, used symptom descriptions outlined in CPP templates to support their decisions to refer patients. They described this as a possibility and appreciated that CPPs emphasized the patients’ need for cohesive care pathways and facilitated smoother patient trajectories between primary and secondary healthcare, as illustrated in the next quote:

Overall, the CPPs are great. / I agree, it has put the patient in focus, which we always strive for, but sometimes you lose that in healthcare and easily slip back into this is mine and this is yours. That’s not the case with CPPs, it’s more focused on the patient in the workflow.

(Group interview only physicians nr 3)

The increased focus on the patient in CPPs clarified the roles within the healthcare trajectories between the PHC unit and the hospital, emphasizing the patient’s central position was perceived as a possibility by the physicians. However, physicians also had to weigh the risks and benefits of referring patients through CPPs. They were mindful of patients with symptoms indicating other serious diseases and expressed concern that the focus on cancer might lead to longer wait time due to lower priority and displacement of these cases. Physicians perceived a worry that patients without suspected cancer could often be overlooked. Therefore, they relied on their clinical experience, along with the CPP descriptions, when prioritizing patients’ need of urgent care through their conviction of what could be cancer.

### Creatively making referrals work when suspecting cancer

This category comprises primarily the physicians’ task of comparing their clinical cancer suspicion with the predetermined set of alarm symptoms constituting the well-founded suspicion in the CPP template (i.e., before making referrals for cancer care). Challenges emerged when physicians clinically assessed patients as potential cases of cancer and had to align their suspicion with the CPP template, and at times chose to refrain from using CPPs. In such cases, they used alternative approaches to address each patient’s needs and concerns, seeking workarounds when the patient’s presentations did not conform to the established template.

Physicians experienced their work to be less complicated when their clinical suspicion of cancer aligned with organ-specific alarm symptoms in the CPP template. This was perceived as a possibility since such alignment facilitated their clinical investigations, as exemplified by the following quote:

With the CPPs, I’m able to turn something that requires a fair amount of thought and make it much less complicated. To be able to assess a clear alarm symptom and decide when, where, and how quickly to act. It’s very nice to have clear decision support tools that say for these symptoms, do this.

(Group interview only physicians nr 2)

Furthermore, physicians acknowledged that CPPs helped accelerate the diagnostic process. Even when symptoms were non-organ specific—such as fatigue, diffuse pain, or unintentional weight loss—a particular CPP could still relieve their work as described in the following quote:

We can refer to a specific pathway to XX (hospital) when we do not have a specific cancer diagnosis in mind. We know there is a suspected malignancy, we observe diffuse symptoms, test results and pathological markers, but we do not know the underlying cause.

(Group interview only physicians nr 3)

Thus, when suspicion aligned with the CPP template physicians recognized this possibility and referrals could be made efficiently, even when the location of the suspected cancer was uncertain.

However, physicians also handled challenging situations where their clinical cancer suspicion did not match the CPP templates. This mismatch was particularly noticeable when they had low suspicion of cancer but still needed to investigate patients presenting with alarming symptoms outlined in CPP templates. In such cases, they worried about overburdening the health system with unnecessary referrals based on symptoms with lower likelihood for cancer, like hematuria, included in CPP criteria. In these situations, both nurses and physicians relied on their clinical cancer suspicion to determine the appropriate next steps in triage and appointment scheduling, as well as the referral pathway and sometimes refrained from using the CPP template. However, less experienced physicians tended to refer more patients through CPPs to avoid missing a potential cancer diagnosis, as exemplified in the following quote:

I’m a bit younger, as an inexperienced physician and early in my specialty training, I tend to refer more patients through CPPs than my supervisor. I’ll say to XX (name of supervisor) that I am concerned this patient may have an underlying malignancy, and then she can respond that it’s more likely a kidney dysfunction. It may be easier to refer more patients through CPPs when you are a little more inexperienced.

(Mixed group interview nr 1)

Physicians with less experience expressed concerns about patients whose symptoms might go unnoticed until the cancer had progressed to more advanced stages. Nurses expressed that even if they were not explicitly involved in making CPP referrals, they did use their clinical experiences and creativity when presenting their clinical suspicions as a reason for booking appointments despite the absence of clear alarm symptoms.

Additionally, physicians experienced that CPPs encompassed a limited and simplistic set of symptoms. They felt it was required to help patients whose symptoms did not align with the CPP template, but were perceived as seriously ill, and find workarounds from the template. This led to an increased workload for the physicians since they handled cases where they strongly suspected cancer based on their clinical examinations and diagnostic tests. Thus, they had to find alternative ways to help the patient and refer them to cancer care as explained in this quote:

If I suspect and assess that the patient may have a serious illness, but it does not follow the CPP template, I won’t refer the patient through CPPs. Instead, I’ll write a regular referral and note suspected malignancy or something similar. In the referral, I’ll describe all my suspicions, what the blood tests and clinical examinations have shown, and the patient’s symptoms… It has a lot to do with the clinical picture of the patient, blood tests, and everything else.

(Mixed group interview nr 1)

Despite the need to work around the CPP template, physicians remained committed to facilitating the patient’s pathway to cancer care. Additionally, nurses and physicians described that they used tailored approaches for patients with multiple comorbidities or those in need of palliative care since it was perceived challenging and not adequate to use CPPs then. In such cases, the patient’s preferences and quality of life were prioritized over other interventions like surgeries, as illustrated in a discussion from a mixed group interview including nurses and physicians:

Some older patients may not want to go through all investigations. They may have to go from one examination to the next and it doesn’t improve their quality of life… / Once the CPPs start, it is difficult to stop it, but the patient may feel that there is not much left to do. /So, when do we stop and decide that it is enough and try to provide relief in what remains and not continue investigating until the very end?

(Mixed group interview nr 2)

For physicians and nurses, it was not always as simple as aligning cancer suspicion with the CPP template and alarm symptoms mentioned in this template and further booking appointments and sending a referral to cancer care when there was a suspicion of cancer. To sum up, physicians and nurses used CPPs and their clinical creativity to ensure appropriate care for patients.

### Sensitively informing about cancer suspicion

This category comprises the last step in the process, PHC providers’ task of informing patients about cancer suspicion when CPP has been initiated. Nurses and physicians in PHC faced that deciding whether and how to tell patients about the suspicion of cancer and undertaking CPPs required balancing honesty with emotional sensitivity.

When PHC nurses and physicians suspected cancer, they aimed to communicate what would happen next clearly and be honest while minimizing potential cancer-related anxiety. Nurses talked about how careful they were not to talk about cancer suspicion, even if they sometimes experienced that the patient’s symptoms aroused such suspicions. Despite their intention to be honest, both nurses and physicians described grappling with the use of the word cancer, fearing it might cause distress, as exemplified in the following quote:

When we inform about CPPs, it can be quite ethically challenging… It’s not easy… We want the patient to show up for the examination, and we want them to have their cell phone ready, but we don’t want them lying awake all night worrying about it.

(Group interview only physicians nr 1)

Informing patients about CPPs due to cancer suspicion was a sensitive and not easy but important opportunity to support. On the one hand, such communication can facilitate faster investigations of possible serious diseases; on the other hand, it may contribute to increased anxiety among patients which often accompanies suspicion of cancer. Nevertheless, they felt determined to ensure that information would be easily understandable for patients, thereby clarifying the intentions of being referred through CPPs. Consequently, nurses described this as a possibility and engaged themselves in adapting CPP information according to the patient’s individual needs and the local context. The focus was to give concise and clear information about the intention of started CPP, through a dialogue between the patient and healthcare provider regarding the next investigation steps. Moreover, physicians also perceived this as a possibility and recognized that providing CPP information could be beneficial and enhance patient satisfaction with healthcare, illustrated in the following quote:

Most of the time the patient is satisfied (with CPP patient information) and it provides a sense of peace of mind when you see that they are grateful afterward since it means less wasted time for the patient’s diagnostic process, and it’s more focused. / You are skillful at using the CPP patient information, always, you always hand out the information.

(Group interview only physicians nr 3)

Patient satisfaction influenced physicians to continue involving patients in their care and to use CPP information as a tool for reassurance and clarity.

At the same time, PHC providers also described challenging situations where they deliberately avoided mentioning cancer. In some cases, providers chose not to hand out CPP information to patients, especially when the likelihood of cancer was low or uncertain, to safeguard the patient. They feared that detailed explanations might cause unnecessary anxiety as discussed by a group of nurses and physicians in the following quote.

No, I rarely hand out CPP information directly. Instead, I just say I’m going to send a referral to the hospital, and they’ll follow up on it… / If we describe CPPs in suspected cancer in detail, then I think it would cause quite a lot of anxiety. / Yes, for example with PSA testing, only four out of ten cases are linked to cancer. / The disadvantage is that this creates a huge vacuum and a lot of worry for the patients.

(Mixed group interview nr 2)

Providers strived to handle the anticipated potential illness-related anxiety, and they were cautious of potentially placing the patient in an uncomfortable situation at home and being unable to provide support. Thus, PHC providers strived to safeguard patients from distressing circumstances that accompany suspicion of cancer. However, they also withheld information about the CPPs.

## Discussion

This study provides insights into Swedish PHC providers’ strategies of handling situations involving patients with potential cancer symptoms following the introduction of CPPs. Although PHC providers have previously assessed cancer suspicions and investigated potential cancer, the introduction of CPPs has enabled a more cohesive workflow across healthcare levels. This is in line with previous research showing that PHC providers perceived CPPs as improving the referral process and contributing to more coordinated cancer care [23]. However, our findings reveal that using CPPs in PHC settings is not a simple task and presents several challenges and possibilities, which are discussed below.

The findings indicate that PHC providers’ application of CPPs depends on balancing standardization with flexibility. This flexibility of a standard like CPP is essential for providers since they need to align CPPs with local practice which enables the practical adoption of standardized practices and the benefit of using these. As Timmerman and Epstein [7] argue, developing standardized practices requires a balance between uniform standards, rigidity and adaptability aligned with local practices. Our findings indicate that even if CPPs are aimed to shorten time to cancer diagnosis the PHC providers occasionally refrain from using CPPs, particularly when patients present low-risk-but-not-no-risk symptoms e.g. diffuse symptoms, or when their clinical presentation does not align with the CPP template. For example, handling such clinical situation as low-risk-but-not-no-risk symptoms is already known as an established challenge for PHC providers [29, 30]. This could be linked to our findings that PHC physicians must rely on their conviction, while simultaneously adapting their clinical assessment to fit CPP criteria which may point to an existing tension between physicians’ clinical reality and applying standards such as CPPs in practice. Others have also noted that providers may resist new ways of working when standards are perceived as too rigid or insufficiently aligned with clinical practice [31, 32]. Previous research further suggests that PHC providers value CPPs for facilitating timely investigations, while simultaneously describing challenges in applying standardized criteria to everyday clinical practice [23]. However, our results indicate that even if using CPPs promise faster investigations, they do not necessarily make the application of CPPs straightforward, particularly when handling low-risk-but-not-no-risk-symptoms.

Additionally, even if standards aim to facilitate clinical decisions and early diagnosis, they do not always function in this way, suggesting that reliance on professional competence based on formal knowledge and clinical experiences may remain particularly important in clinical encounters. Our results indicate that assessments of urgency seem to rely directly on professional competence, using professional intuition and conviction rather than simply relying on descriptions in the CPP template. A tension between reliance on professional competence, both one’s own and that of colleagues’, and the use of standardized tools became apparent. Thus, trusting in each other’s professional competencies remained important for interpreting the seriousness of symptoms despite the use of CPP templates. Similarly, Van Dongen et al. [33] have shown that trust among professionals in PHC promotes their actions beyond their respective professional responsibilities. Hämel and Vössing [34] also note that PHC nurses and physicians who trust one another and combine their professional competencies in collaboration can provide healthcare through teamwork on more equal terms, despite potential professional tensions. Clinical competence is described as something patients appreciated and expected from physicians and also experienced it as improving medical assessments and examinations by physicians, as well as patient satisfaction and trust in physicians [35].

Interestingly, our results also showed that informing patients about the initiation of a CPP elicited an embedded tension between clearly talking about cancer suspicions and protecting patients by avoiding too much information and using professional sensitivity. This also revealed a tension between being honest and transparent for patients’ sake and at the same time limiting information to only what is necessary in an attempt to diminish the burden of a potential cancer diagnosis. Additionally, it highlights the tension between the professional obligation to inform and the desire to avoid causing unnecessary harm or applying a paternalistic view. Even if being careful when informing seems adequate, limiting information could also be seen as using professional power. Reflecting on patients’ will to know and capability to manage information is important and necessary. In such situations, PHC providers must balance the need to communicate uncertainty with the responsibility to provide clear guidance regarding the next steps following a cancer suspicion [36]. This approach aligns with research suggesting that attentive listening and clear communication can enhance patient trust and understanding [37]. Solbjør et al. [38] found that patients feel reassured when informed about CPPs, as it provides clarity and prioritization within the health system. However, previous research has noted that limiting and avoiding information may also lead to miscommunication and delays in diagnosis [39, 40]. Informing but individually adapting information is necessary if we want to see patients as capable people to manage their health. Providers must therefore balance between minimizing distress and ensuring patients are adequately informed. While most providers follow the requirement to inform patients about CPPs, withholding detailed information can accidentally contribute to anxiety and uncertainty.

### Strengths and limitations of the research design

The GTM proved to be a valuable approach for openly and flexibly exploring the research aim and understanding our findings related to the CPP introduction. Additionally, the multidisciplinary research team, consisting of members from nursing, social and medical sciences, and the gender-equal research group, combined knowledge from PHC and cancer research to provide diverse perspectives during data collection and analysis. Northern Sweden presents a significant challenge for PHC provision due to its widely dispersed population across vast geographical areas, with few cities. To represent this setting, we included a variety of PHC units, including both small and large-sized units in urban and sparsely populated areas, as well as participants with different ages and genders with diverse professional backgrounds and varying levels of experience. This enabled us to capture a broad range of perspectives. Furthermore, we collected data from both small and large groups, with nurses and physicians participating either together or separately. Additionally, the composition of the interview groups can be a limitation since it may have influenced our results. Homogeneous groups may facilitate discussion of profession-specific experiences, whereas mixed groups may stimulate interaction across professional perspectives but also introduce hierarchical dynamics that can influence participants’ willingness to express divergent views [41]. However, during the analysis, we checked that codes and emerging categories were present and supported by the data from all interview-groups, even if some aspects were more prominent in some groups due to the professional homogeneity in the group. One limitation encountered during data collection was that we had to consider the daily workload and time constraints of PHC providers. This resulted in limitations of 45–60 min for group interviews and led to restrictions for some PHC providers to participate. Consequently, some perspectives may not have been represented in the data. Although interview duration varied between 37 and 56 min, all interviews contributed codes and insights that informed the development of the final categories. Nevertheless, the variation in groups composition presented opportunities to explore both profession-specific perspectives and interactions between professional groups, while also requiring consideration of how group dynamics and professional hierarchies may have shaped participants’ contributions.

Additionally, our findings are based on participants’ accounts of their experiences and perceptions of CPP use in PHC without observations of clinical practice. The findings may also be influenced by professional perspectives in the findings as physicians have the formal responsibility for CPP referrals, experiences related to CPP initiation and referral decisions may be more reflected in the findings. Lastly, the interviewers provided both internal and external perspectives to ensure as rich and diverse data collection as possible. Both perspectives were crucial to addressing our preconceptions and thereby ground emerging categories in the actions and meanings of the participants. The multidisciplinary backgrounds of the research team, including nursing, medicine, and social sciences, increased opportunities for diversity in the interpretation of the data. These differing perspectives contributed to a broader understanding of participants’ experiences but also required continuous reflexive discussions regarding assumptions and alternative interpretations throughout the analytic process. Regular discussions within the research team were therefore used to critically examine how professional and disciplinary perspectives might shape emerging interpretations and category development.

## Conclusion

In conclusion, our findings underscore a process to handle challenges and possibilities when using CPPs in PHC in northern Sweden, but also rely on their own clinical competence and creativity to prioritize patients’ needs and allocate healthcare resources appropriately when investigating cancer suspicions with sensitivity. Standardized practices such as CPPs were perceived as presenting some possibilities for facilitating a faster cancer diagnostic pathways, while also creating challenges to use them. CPPs appeared as more likely adopted when they aligned with PHC providers’ clinical assessment. However, challenges arose when presented symptoms and PHC providers’ clinical assessments regarding cancer suspicion did not align with CPP templates. This highlights the need of involving PHC providers in refining CPPs in Sweden to better reflect and integrate PHC’s perspective and clinical practice. Furthermore, it is essential to sensitively and honestly inform patients about potentially suspected cancer and the next steps in a specific CPP. PHC providers need to improve strategies to honestly inform patients and ensure that they adequately understand the CPP process. Developing communication strategies that balance clear information without scaring patients is crucial to maintaining trust and engaging patients in healthcare processes.

## Implications for policy and practice

Our study highlights the importance of incorporating the experiences of PHC providers into the ongoing development and refinement of CPPs. Policymakers should acknowledge the diversity of clinical practices within PHC and promote standardized practices that support flexibility. It is crucial to empower providers to assess each patient based on individual needs, while still aligning standardized templates like CPPs to improve the quality and efficiency of care. Achieving this balance requires CPPs to be adaptable to varying clinical contexts—including situations where symptoms do not neatly fit into existing templates—and to allow providers the flexibility to act on their professional competencies. Additionally, actively involving PHC providers in the evaluation and revision of CPPs through feedback loops is vital to ensure that these integrated care pathways remain responsive to clinical practices. Creating an environment of continuous learning through dialogue and collaborative refinement can support the adoption of CPPs and ultimately facilitate patients access to care.

## Supplementary Information

Below is the link to the electronic supplementary material.

Supplementary Material 1

Supplementary Material 2

## Data Availability

The datasets generated and/or analyzed during the current study are not publicly available due to the data including information that could compromise research participants’ privacy and consent but are available from the corresponding author on reasonable request.

## Declarations

### Guideline statement

All methods were carried out in accordance with relevant guidelines and regulations.

## Abbreviations

CPP: Cancer Patient Pathways
GTM: Grounded Theory Method
PHC: Primary Healthcare
