# Supplementary material for: Primary healthcare providers’ strategies of handling patients presenting with potential cancer symptoms following the introduction of standardized cancer patient pathways in Sweden
Source: BMC Prim Care. 2026 Oct 10;27:368. doi: 10.1186/s12875-026-03600-2 (PMC13670806; doi:10.1186/s12875-026-03600-2)
Supplement: Supplementary file 2 [file 12875_2026_3600_MOESM2_ESM.pdf]

Appendix 1

1. Can you tell me what you do when you meet with or talk to patients about their complaints/symptoms that you find alarming (alternative: red flags/warning flags)?

a) Do you have anything specific in mind and if so, what?
b) Do you have any routines or any special methods that you use when you meet with or talk to these patients?
c) How do you do it on the phone or in-person at the clinic?

2. Have you experienced any changes since the introduction of CPP in your system or at your healthcare center for how you handle patients? In what ways?

3. Do you experience that the introduction of CPP has affected how you yourself handle patients? In what ways?

4. Has the introduction of CPP affected the way you handle patients in relation to other professions/each other?

5. In what ways do you experience that CPP facilitates how you resolve situations when patients tell you about their complaints/symptoms that you find alarming?

6. In what ways do you feel that CPP makes it more difficult to resolve situations when you meet patients who you suspect need to see a doctor because you cannot rule out cancer?

7. CPP has now been introduced and your experience is [SUMMARY OF WHAT THEY HAVE SAID SO FAR]. Can you tell us how you have been involved in that work?

8. Concerning the introduction of CPP, has anyone (from the management in the county councils or at the healthcare center) asked you how you want CPP to work at your healthcare center? How did it go?

9. CPP has now been around for a while and we are wondering if you could now reflect on how you handled patients before CPP came along and how you do it today?

10. Is there anything that you see at your healthcare center or in general that could have been done differently?

Intervjuguide för fokusgruppdiskussioner på primärvårdsnivå
Läkare/Sjuksköterska Vårdcentral

Forskningsfråga: How are care organizations responding to the implementation of CPPs at the primary, specialist and even policy level

Man har en inledning om SVF före och så går man över till frågorna.

1. Kan ni berätta hur ni gör när ni möter eller pratar med patienter som berättar om sina besvär /symptom som ni tycker alarmerar (alternative: röda flaggor/varningsflagor)?
    a. Tänker ni på något specifikt och i så fall hur då?
    b. Har ni några rutiner eller något särskilt sätt som ni använder er av när ni möte eller pratar med dessa patienter?
    c. Hur gör ni i telefon respektive mottagning?
2. Upplever ni att införande av SVF inneburit någon förändring i systemet eller på er vårdcentral för hur ni handlägger patienter? På vilka sätt?
3. Upplever ni att införande av SVF påverkat hur ni själva handlägger patienter? På vilka sätt?
4. Har införande av SVF påverkat sättet på hur ni i relation till andra professioner/varandra handlägger patienter?
5. På vilka sätt upplever ni att SVF underlättar hur ni löser dessa situationer när patienter berättar om sina besvär /symptom som ni tycker alarmerar?
6. På vilka sätt upplever ni att SVF försvårar hur ni löser dessa situationer när ni möter patienter som ni misstänker behöver träffa läkare eftersom ni inte kan utesluta cancer?
7. Nu har SVF blivit infört och er erfarenhet är [SAMAFATTA VAD DE HAR SAGT HITTILLS]. Kan ni berätta hur har ni varit involverade i det arbetet?
8. Har någon (från ledningen i landstingen eller på VC) i samband med införande av SVF frågat er hur ni vill få det att fungera på er vårdcentral? Hur gick det till?
9. Nu har SVF funnits ett tag och då funderar vi om ni nu skulle kunna kan reflektera över hur ni hanterade patienter innan SVF kom och hur ni gör det idag?
10. Är det något som ni ser på er HC eller generellt som kunde har gjorts på annat sätt?
